# Local Anesthetic-Induced Central Nervous System Toxicity during Interscalene Brachial Plexus Block: A Case Series Study of Three Patients

**DOI:** 10.3390/jcm10051013

**Published:** 2021-03-02

**Authors:** Daniel Spitzer, Katharina J. Wenger, Vanessa Neef, Iris Divé, Martin A. Schaller-Paule, Kolja Jahnke, Christian Kell, Christian Foerch, Michael C. Burger

**Affiliations:** 1 Institute of Neurology (Edinger Institute), University Hospital Frankfurt, Goethe University, 60528 Frankfurt, Germany; spitzer@med.uni-frankfurt.de; 2 Department of Neurology, University Hospital Frankfurt, Goethe University, 60528 Frankfurt, Germany; iris.dive@kgu.de (I.D.); martin.schaller@kgu.de (M.A.S.-P.); kolja.jahnke@kgu.de (K.J.); c.kell@em.uni-frankfurt.de (C.K.); foerch@em.uni-frankfurt.de (C.F.); 3 Institute of Neuroradiology, University Hospital Frankfurt, Goethe University, 60528 Frankfurt, Germany; katharina.wenger@kgu.de; 4 Department of Anesthesiology, Intensive Care Medicine and Pain Therapy, University Hospital Frankfurt, Goethe University, 60590 Frankfurt, Germany; vanessa.neef@kgu.de; 5 Dr. Senckenberg Institute of Neurooncology, University Hospital Frankfurt, Goethe University, 60528 Frankfurt, Germany; 6 University Cancer Center Frankfurt (UCT), University Hospital Frankfurt, Goethe University, 60590 Frankfurt, Germany; 7 Frankfurt Cancer Institute (FCI), 60596 Frankfurt, Germany; 8 German Cancer Consortium (DKTK), Partner Site Frankfurt/Mainz, 60590 Frankfurt, Germany

**Keywords:** interscalene brachial plexus block, local anesthetic, CNS toxicity, toxic hemisphere syndrome, neurological outcome

## Abstract

Local anesthetics are commonly administered by nuchal infiltration to provide a temporary interscalene brachial plexus block (ISB) in a surgical setting. Although less commonly reported, local anesthetics can induce central nervous system toxicity. In this case study, we present three patients with acute central nervous system toxicity induced by local anesthetics applied during ISB with emphasis on neurological symptoms, key neuroradiological findings and functional outcome. Medical history, clinical and imaging findings, and outcome of three patients with local anesthetic-induced toxic left hemisphere syndrome during left ISB were analyzed. All patients were admitted to our neurological intensive care unit between November 2016 and September 2019. All three patients presented in poor clinical condition with impaired consciousness and left hemisphere syndrome. Electroencephalography revealed slow wave activity in the affected hemisphere of all patients. Seizure activity with progression to status epilepticus was observed in one patient. In two out of three patients, cortical FLAIR hyperintensities and restricted diffusion in the territory of the left internal carotid artery were observed in magnetic resonance imaging. Assessment of neurological severity scores revealed spontaneous partial reversibility of neurological symptoms. Local anesthetic-induced CNS toxicity during ISB can lead to severe neurological impairment and anatomically variable cerebral lesions.

## 1. Introduction

Peripheral nerve blocks are used for regional anesthesia. They are an effective, highly standardized and routinely used method to provide supplementary analgesia in a surgical setting [1], particularly for providing high-level analgesic treatment in the immediate postoperative period [2,3,4,5]. For a peripheral nerve block, the local anesthetic is injected near a specific nerve or bundle of nerves. The brachial plexus block is one of the most commonly used regional anesthetic procedures for upper limb surgery [6] and is often combined with general anesthesia [7]. Depending on the location and type of surgery planned, as well as patient-specific habitus, there are several technical approaches to block the brachial plexus, including the axillar, the interscalene, the supraclavicular, and the infraclavicular approach [8,9].

The interscalene brachial plexus block (ISB) as well as the placement of a perineural catheter within the interscalene space follow a standardized technical approach. These are straight-forward procedures associated with an incidence of short- and severe long-term complications of only 0.4% [10]. Two technical approaches are typically used to determine the injection point for ISB, including nerve stimulator-guided ISB (NS-ISB) [11] and ultrasound-guided ISB (US-ISB) [12], or a combination of both [13]. NS-ISB has been reported to have a similar efficacy, success rate, and rate of postoperative neurological symptoms compared to US-ISB [14]. Acute adverse events associated with ISB include systemic toxicity, cardiac arrest, pneumothorax, and respiratory distress [15,16,17,18,19].

Long-term neurological deficits commonly attributable to nerve injury-induced peripheral neuropathies however are very rare and were reported to occur in 0.029% of patients who received an ISB [20]. However, transient nerve blocks are very common, occur in up to 100% of patients and are mostly volume- and technique-dependent [21]. Hemidiaphragm palsy due to a phrenic nerve block occurs in 15 to 100% [21,22]. Horner’s syndrome due to a blockade of the sympathetic stellate ganglion occurs in 8 to 12% [21,23]. Recurrent laryngeal nerve palsy presenting with symptoms such as hoarseness, dysphonia and dysphagia occurs in approximately 0.8 to 1.3 of patients [24,25].

There are several reports of severe and often fatal toxicity induced by accidental intraspinal injection of local anesthetics during ISB causing total spinal anesthesia [26,27]. However, to the best of our knowledge, there are only few clinical reports describing cerebral symptoms including convulsions, behavioural changes, or transient aphasia in a temporal relationship with ISB [10,28,29,30,31]. Moreover, there is no dedicated report on patients suffering from persistent (long-term) neurological symptoms traced back to brain lesions presumably caused by the local anesthetics used for ISB. This work describes neurological complications of local anesthetic procedures, the severity of symptoms, neuroradiological findings and clinical outcome in a sample of three patients that were treated in the course of three years.

## 2. Experimental Section

Ethical approval of the study was granted by the institutional Review Board of the Ethical Committee at the University Hospital Frankfurt (project number: 20–699). Outcome data were gathered after the patients signed an informed consent form specifically authorizing prospective data collection.

### 2.1. Patients

In this case series, we report on three patients who were admitted to the intensive care unit (ICU) of the Department of Neurology at the tertiary care hospital of the Goethe University between November 2016 and September 2019 with initially suspected perioperative stroke following ISB for arthroscopic rotator cuff repair performed at external hospitals.

Patient 1 was a 52-year-old male with a medical history of multiple sclerosis, who underwent arthroscopic rotator cuff repair due to a spontaneous complete rupture of the left supraspinatus tendon. The positioning of the patient applied during surgery was the beach chair position. Preoperative blood pressure (BP) was 135/80 mm Hg; heart rate (HR) 66 beats per minute (bpm); oxygen saturation (SpO_2_) values between 98% and 100%. For premedication, the patient received orally administered midazolam. Single-injection ISB was performed prior to induction of anesthesia and intubation. Total intravenous anesthesia (TIVA) was maintained with propofol and sufentanil. For left ISB, the patient initially received single-dose injections of 1% lidocaine (20 mL) and 0.75% ropivacaine (20 mL) followed by a second single-dose injection of 0.75% ropivacaine (10 mL) three hours after initial local anesthetic injections and 15 min prior to termination of TIVA, and extubation. Safety checks prior to injection did not show any aspiration of blood or cerebrospinal fluid. Adverse cardiac or neurologic symptoms were not recorded. Advancement and placement of the catheter was performed in PNS technique using a stimulating catheter. An effective ISB was confirmed by physical examination prior to surgery. BP was measured every 5 min with a noninvasive cuff. The patient underwent an uneventful operative rotator cuff repair under the ISB and intravenous anesthesia. Intraoperatively, recorded systolic blood pressure (SBP) levels of 100–110 mmHg were interrupted by SBP levels of ≥90 and <100 mmHg for a maximum of 90 min due to the application of controlled hypotension. Administration of antihypertensive and/or vasoconstrictor drugs was not recorded. SpO_2_ (98% to 100%), and end-tidal CO_2_ (ETCO_2_) values (30% to 34%) were normal throughout the case. The duration of surgery was 170 min. After termination of TIVA, and extubation in the operating room (OR), the patient remained in a severely impaired conscious state. In the postanesthesia care unit the patient showed a sustained severe impairment of consciousness, and neurological examination by a neurologist additionally revealed aphasia, multi-modal neglect to the right, left-sided gaze preference, dysphagia, severe right-sided central facial palsy, severe right-sided hemiparesis graded as 2/5 according to the Medical Research Council (MRC) scale for muscle strength, right-sided positive pyramidal tract signs, and right-sided hemihypesthesia. These symptoms were further accompanied by complex behavioural manifestations (anxiety, vocalization, aggression) and facial motor movements suggesting complex focal seizures. Due to vomiting with pulmonary aspiration, the patient underwent an emergency intubation. Brain imaging four hours post-surgery did not detect any cerebral lesions.

Patient 2 was a previously healthy, 52-year-old male with a spontaneous complete rupture of the left supraspinatus tendon. The patient received an arthroscopic rotator cuff repair, and a single-injection ISB combined with TIVA was selected as the anesthetic strategy. Preprocedural BP was 130/60 mm Hg; HR 66 bpm; SpO_2_ values 99–100%. After ultrasound- and PNS-guided placement of a left ISB, the brachial nerve block was performed by injecting 1% lidocaine (10 mL) followed by 0.375% ropivacaine (10 mL). There was a negative aspiration test for blood and cerebrospinal fluid prior to injection. Physical examination revealed an effective interscalene block. Adverse cardiac or neurologic symptoms were not recorded. After induction of anesthesia, and intubation, TIVA was maintained with propofol and sufentanil. BP was measured every 5 min with a noninvasive cuff. The patient underwent an uneventful operative rotator cuff repair under the ISB and intravenous anesthesia. Intraoperatively, recorded SBP levels of 100–120 mmHg were interrupted by SBP levels of ≥90 and <100 mmHg for a maximum of 35 min due to the application of controlled hypotension. Administration of antihypertensive and/or vasoconstrictor drugs was not recorded. SpO_2_ (98–100%) and ETCO_2_ values (34–37%) were normal throughout the case. The duration of surgery was 56 min. After termination of TIVA, and extubation in the OR, the patient presented with impaired consciousness and aphasia, a left-sided gaze preference, dysphagia, mild-to-moderate right-sided central facial palsy, right-sided hemiplegia, and right-sided positive pyramidal tract signs. Brain imaging four hours post-surgery did not detect any cerebral lesions.

Patient 3 was a 71-year-old male with partial tear of the left rotator cuff who received an arthroscopic rotator cuff repair. The patient had an unremarkable medical history. The positioning of the patient applied during surgery was the beach chair position. An ISB combined with TIVA was planned as the anesthetic strategy. Left ISB was performed by injecting a single-dose of 1% lidocaine (20 mL) and 0.75% ropivacaine (20 mL) followed by continuous interscalene infusion of 0.2% ropivacaine at 6–8 mL/h. There was a negative aspiration test for blood and cerebrospinal fluid prior to injection. Advancement and placement of the catheter was performed in PNS technique using a stimulating catheter. Physical examination prior to surgery confirmed an effective interscalene block. Adverse cardiac or neurologic symptoms were not recorded. For the entire duration of surgery, the patient remained hemodynamically stable and the case proceeded uneventfully under general anesthesia. Immediately after termination of TIVA, and extubation in the OR, the patient presented a considerable delay of waking reactions and aphasia, left-sided gaze preference, severe right-sided central facial palsy, and right-sided severe hemiparesis graded as 1/5 according to the MRC scale for muscle strength. These symptoms persisted during postoperative monitoring in the postanesthesia care unit. Brain imaging three to four hours post-surgery did not detect any cerebral lesions.

### 2.2. Definitions

Local anesthetic-mediated central nervous system (CNS) toxicity was strongly suspected in case of new postoperative CNS symptoms including impaired consciousness or focal neurological deficits [32]. Focal neurological signs were categorized as assumingly ascribed to brain lesions (i.e., in case of cortical signs) or spinal lesions or peripheral nervous system lesions, as unifocal or multifocal, and as transient or persistent. As patients were admitted to our intensive care unit initially with suspected diagnosis of perioperative stroke, the National Institutes of Health Stroke Scale (NIHSS) was used to determine the severity of neurological symptoms [33]. For the assessment of patient status and severity of disease during ICU treatment the Simplified Acute Physiology Score II (SAPS II) was measured 24 h after admission [34]. Patients’ level of consciousness impairment was assessed at least three hours after cessation of anesthesia using the Glasgow Coma Scale (GCS) and defined as no, mild, moderate, severe consciousness impairment or coma [35,36].

Seizure activity was defined as continuous or recurrent rhythmic focal or generalized spikes, sharp waves, spike waves, or rhythmic waves changing in amplitude, frequency, and/or spatial distribution as evidenced by EEG [37]. Non-convulsive status epilepticus was diagnosed according to the Salzburg Consensus Criteria for diagnosis of Non-Convulsive Status Epilepticus (SCNC) [38].

### 2.3. Diagnosis and Treatment

Upon admission, ICU management focused on symptomatic life-supporting treatment and seizure control. Clinical examination and laboratory findings were performed in order to rule out common causes for impaired consciousness such as hyperthermia, hypo- or hyperglycemia, hypo- or hypercapnia, anemia, sepsis or other metabolic disturbances. Patients with clinical signs of status epilepticus were managed according to the guidelines for the evaluation and management of status epilepticus [39].

In the emergency setting, one patient received magnetic resonance imaging (MRI), while two patients had already initially received a computer tomography (CT) scan. However, MRI was subsequently performed in all patients. CT or MRI were combined with CT or MR angiography in all patients to exclude ischemic stroke secondary to large vessel stenosis or occlusion as well as cerebral vein or sinus thrombosis. Cerebrospinal fluid was examined in patients to rule out an acute manifestation of autoimmune- or pathogen-mediated encephalitis when deemed appropriate by the attending physicians. Qualitative tests for toxic agents or medications associated with seizures and other symptoms of toxic encephalopathy were performed at the discretion of the attending physicians.

## 3. Results

### 3.1. On-Scene Clinical Presentation

Patient characteristics are summarized in Table 1. In all three patients, hemisphere syndrome following ISB on the left side was associated with moderate or severe impairment of consciousness according to the Glasgow Coma Scale (median GCS score 8, Table 2) and severe multifocal neurological deficits with a median baseline NIHSS score of 24 (Table 2). Neurological symptoms of left hemisphere syndrome included global aphasia, left-sided gaze preference and severe right-sided hemiparesis or hemiplegia in all patients, while patient 1 also showed a multi-modal neglect to the right. Patient 1 and patient 2 additionally presented with dysarthria, dysphagia and right-sided positive pyramidal tract signs. One out of three patients (patient 1) additionally showed complex behavioural manifestations including anxiety, vocalization and aggression, and motor movements suggesting complex focal seizures. All three patients presented with clear CNS signs attributable to cortex dysfunction (i.e., aphasia, hemineglect). Therefore, and due to the perioperative onset, the initially suspected diagnosis at time of treatment initiation was perioperative stroke in all three patients.

### 3.2. Neuroimaging

**Figure 1:**
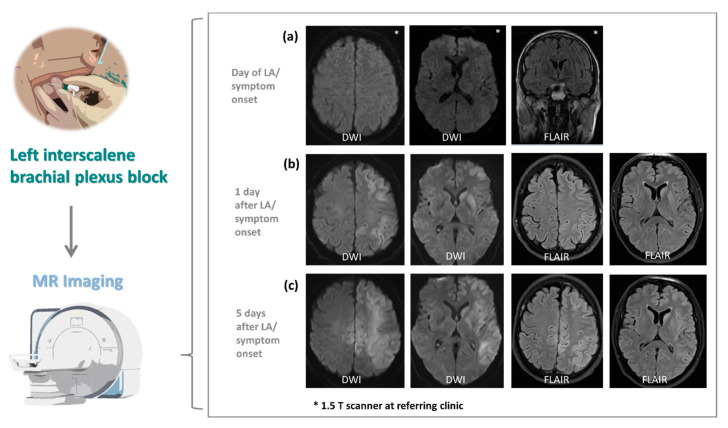
Magnetic resonance imaging (MRI) results of patient 2 admitted to the neurological intensive care unit with suspected stroke following interscalene brachial plexus block (ISB). (**a**) Diffusion-weighted imaging (DWI) and fluid-attenuated inversion recovery (FLAIR) sequence as part of the standard MRI screening protocol (DWI/ADC, T2 TSE, FLAIR, arterial TOF-MR-angiography) designed for patients with suspected stroke does not show cerebral lesions on the day of local anesthesia (LA) and symptom onset. (**b**) DWI shows restricted diffusion with corresponding FLAIR hyperintensities in the left caudate nucleus and putamen, as well as cerebral cortex of the frontal, temporal and parietal lobe one day after LA. (**c**) Five days after LA, DWI demonstrates increasing areas of restricted diffusion with corresponding FLAIR hyperintensities. Changes now additionally involve left deep subcortical white matter of the frontal, temporal and parietal lobe. Note new cortical and subcortical changes in the superior margin of both hemispheres, as well as the globus pallidus, hippocampus and the cerebral crus on the left side. No signal changes in T1-weighted MRI. Unless indicated otherwise, all examinations were performed on a 3 T whole-body magnetic resonance system (MAGNETOM Skyra; Siemens Healthcare, Erlangen, Germany).

**Figure 2:**
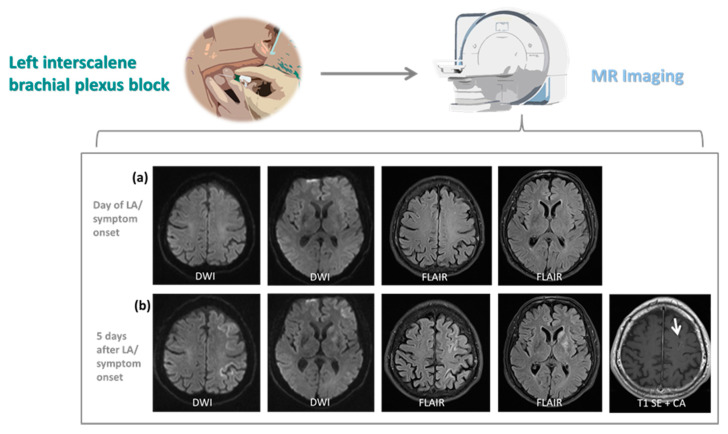
Magnetic resonance imaging (MRI) results of patient 3 admitted to the neurological intensive care unit with suspected stroke following interscalene brachial plexus block (ISB). (**a**) Diffusion-weighted imaging (DWI) and fluid-attenuated inversion recovery (FLAIR) sequence as part of the standard MRI screening protocol used for patients with suspected stroke (DWI/ADC, T2 TSE, FLAIR, T2*, arterial TOF-MR-angiography) does not reveal cerebral lesions on the day of local anesthesia (LA) and symptom onset. (**b**) Five days after LA, DWI shows restricted diffusion with corresponding FLAIR hyperintensities in the basal ganglia, including caudate nucleus, putamen and globus pallidus, as well as cerebral cortex of the frontal and medial superior gyrus, and central and post central gyrus of the left hemisphere. Diffuse cortical enhancement (arrow) in the left frontal lobe is detected in contrast-enhanced T1-weighted MRI. All examinations were performed on a 3 T whole-body magnetic resonance system (MAGNETOM Skyra; Siemens Healthcare, Erlangen, Germany).

The patients underwent cerebral imaging including CT and MRI according to protocols designed for patients with suspected stroke. However, no abnormal imaging findings were observed in standard MRI protocol (DWI/ADC, T2WI TSE, FLAIR, T2*, arterial TOF-MR-angiography) on the day of ISB and symptom onset in all patients (Figure 1a, patient 2; Figure 2a, patient 3; patient 1 not shown).

Two out of three patients (patient 2 and 3) developed findings on MRI on day one and/or day five post ISB. More specifically, these patients showed T2/FLAIR hyperintensities with ADC restriction of cortical grey matter and basal ganglia of the left hemisphere (Figure 1b and Figure 2b). MRI of patient 2 later showed additional involvement of deep subcortical white matter, hippocampus and the cerebral crus corresponding to the side of ISB, and involvement of cortical grey matter of the superior margin of the opposite hemisphere (Figure 1c). While in patient 3 abnormal signal intensities corresponded to middle cerebral artery (MCA) vascular territory only (Figure 2b), patient 2 showed abnormalities in multiple territories including MCA, anterior cerebral artery (ACA) and posterior cerebral artery (PCA) on the side of ISB as well as ACA in areas of leptomeningeal collaterals on the opposite hemisphere (Figure 1b,c). Contrast-enhanced T1-weighted MRI revealed diffuse cortical enhancement in patient 3 five days after ISB (Figure 2b). These findings demonstrate an early involvement of grey matter. With no application of contrast agent within the first 4 days after symptom onset, initial cytotoxic and later vasogenic edema, possibly followed by cell death, can only be speculated upon. While MRI showed a pattern of vascular distribution on the side of ISB, vessel occlusion was not detected at any time on MR angiography.

### 3.3. Clinical Management and Neurological Outcome at Discharge

Total length of hospital stay ranged from 9 to 19 days (Table 1). All patients were in poor clinical condition according to SAPS II 24 h after admission (median score 41, Table 2). Extended intensive care treatment including mechanical ventilation due to aspiration pneumonia was needed in one patient (patient 1, Table 1). EEG demonstrated persistent moderate to severe slowing in the affected hemisphere in all three patients (Table 1), indicating underlying focal cerebral dysfunction. Clinical seizures occurred in two patients (patients 1 and 2), which progressed to non-convulsive status epilepticus in one patient (patient 2, Table 1).

Patients 1 and 2 showed partial, and patient 3 full reversibility of slow wave activity in the affected hemisphere. At the time of hospital discharge, seizure activity (both on EEG and clinically) was not observed in any patient (Table 1). Assessment of NIHSS scores revealed spontaneous partial reversibility of the left hemisphere syndrome in all three patients; median NIHSS score improved from 24 on admission to 10 at discharge (Table 2). Moreover, patients regained full recovery of consciousness during their hospital stay; median GCS scores improved from 8 on admission to 14 at discharge (Table 2). Despite partial reversibility of neurological symptoms, the assessment of functional outcome revealed persistent moderate to severe disability in all patients with a median Glasgow Outcome Scale (GOS) score of 3 at discharge from our hospital (Table 2).

### 3.4. Follow-Up and Outcome

Following acute care, all patients underwent an inpatient rehabilitation treatment. Unfortunately, patient 2 passed away during rehabilitation due to a complicative pulmonary artery embolism. The functional outcome of patients 1 and 3 improved during their rehabilitative stay of 12 weeks. Patients 1 and 3 were invited for clinical re-evaluation after 28 and 47 months, respectively, and had achieved significant improvement of neurological functions (Table 2). Patient 1 showed a right-sided central facial paresis, mild paresis of the right arm and reduced fine motor skills of the right hand. The muscular reflexes of the right arm and leg were increased, while the plantar reflex was negative. Furthermore, the patient presented with a moderate gait instability. Patient 1 regained autonomy in daily activities, but was not able to resume his previous academic professional activity due to difficulties in concentration. He reported a diminished resilience, difficulties remembering names and occasional word retrieval difficulties, which however was not apparent during clinical examination. Similarly, patient 3 reported occasional difficulties finding the right words during conversation, which was also not apparent during clinical examination. In patient 3 finger flexion was mildly paretic with fine motor skills of his right hand reduced. The muscular reflexes of the right arm were increased. Patient 3 also presented with a mild gait instability, which however did not impede him to return to part-time professional activity requiring walking long distances. Both patients 1 and 3 reported that their condition showed no further improvement since discharge from in-patient rehabilitation treatment despite subsequent out-patient physiotherapy.

## 4. Discussion

Large prospective studies have demonstrated that peripheral nerve block is an effective method of providing supplementary analgesia in the surgical setting. The various techniques reported are associated with a low incidence of acute complications that are mostly of transient nature. Nevertheless, there are a few reports describing persistent neurological symptoms after peripheral nerve block [40,41,42], indicating a variable extent of neurological deficits persisting beyond hospital discharge.

Here, we describe to the best of our knowledge for the first time in detail three cases of severe stroke-like symptoms following ISB which we attribute to toxic effects to the brain caused by lidocaine and ropivacaine. We initially suspected a perioperative ischemic stroke in all three patients. There are several reports of patients who suffered cerebral ischemia which were linked with shoulder surgery, and especially for the beach chair position, which is used as the standard position [43,44,45]. However, several observations strongly argue against an ischemic etiology: DWI positive lesions were observed in none of the three patients at the day of symptom onset, when all three patients exhibited a pronounced hemisphere syndrome. While DWI negativity indeed does not rule out ischemia, the majority of DWI negative strokes are small lacunar strokes or located in the posterior circulation [46,47,48] with an NIHSS score of less than 5 [49]. Patients 2 and 3 developed restricted diffusion only in follow-up imaging indicative for cytotoxic edema. These diffusion restrictions followed a pattern of vascular distribution. Nevertheless, no vessel occlusions were observed, while in patients with a severe hemisphere syndrome caused by a major ischemic stroke, imaging performed in the acute situation often reveals the occlusion of intracerebral vessels [50,51]. Also the presence of predominant grey matter lesions of several vascular territories in follow-up MR imaging on the other hand were not indicative of ischemic stroke. Interestingly, the hemisphere syndrome in our three patients was on the same side where the ISB was applied. During the observation period, we did not observe any patients with a hemisphere syndrome contralateral to a recently applied ISB. While none of the arguments described above can definitely rule out an ischemic etiology, the synopsis of the arguments presented above make an ischemic etiology seem implausible.

The unilaterality of brain lesions and the absence of hemispheric watershed infarction also argued against a generalized hypoxic brain damage/hypotension-induced cerebral ischemia [52], especially as in none of the patients a sudden and steep intraoperative blood pressure drop was reported. Hemodynamic stability was maintained in all the patients during surgery in the beach chair position with recorded systolic blood pressure (SBP) levels of ≥ 100 mmHg. In patient 1 and 2 a controlled hypotension was applied, but maintaining SBP levels between 90 and 100 mmHg without need for administration of vasoconstrictor drugs. Furthermore, vascular stenotic lesions of the carotid artery and congenital/acquired variations of circle of Willis anatomy were ruled out, which both might render a patient more vulnerable to hypotension during surgery in the beach chair position.

In all patients, the effectiveness of ISB prior to general anesthesia had been confirmed clinically, which suggests that sufficient amounts of the local anesthetics were injected to the intended location. We neither were able to detect a hematoma of the soft tissue surrounding the extracranial arterial vessels, which are in close proximity to the injection trajectories of the interscalene approach [53], nor could we identify signs of arterial dissection in the angiograms verifying unintentional vessel puncture. However, it is plausible that accidental intravascular injection of local anesthetics was the causative factor for this complex symptomatology. Of note, test aspirations fail to identify an intravascular needle placement in up to 2% of patients [54]. Additionally, even a small amount of local anesthetic injected unintentionally within an extracranial artery supplying the brain can result in concentrations sufficient to induce CNS toxicity [55]. Our conclusion is further corroborated by MRI scans, showing abnormal MRI signal intensities in the corresponding cerebral vascular territories on the side of ISB in two patients (patients 2 and 3), strongly supporting the hypothesis of an arterial blood flow-mediated local anesthetic CNS toxicity. MRI showed a pattern of vascular distribution primarily on the side of ISB in patient 2 and 3, and additional lesions in areas of leptomeningeal collaterals on the contralateral hemisphere in patient 2. Importantly, vessel occlusion in these patients was not detected at any time. We observed neurological deficits anatomically attributable to T2/FLAIR hyperintensities with ADC restriction of cortical grey matter and basal ganglia, showing an early involvement of grey matter of the affected hemispheres. Therefore, we assume initial cytotoxic and later vasogenic edema possibly followed by cell death.

In summary, there is strong radiological evidence for neurotoxicity of local anesthetics for which the mechanism is apoptotic cell death [56], implying arterial distribution of toxic concentrations of local anesthetic in these patients. The neuroradiological findings are in line with imaging features characterized in one case report describing a previously healthy adolescent suffering from a status epilepticus following local anesthesia [55]. In this female patient, presumed accidental intravascular injection of lidocaine during inferior alveolar nerve block was associated with bilateral hyperintense T2 cortical lesions with restricted diffusion on DWI and ADC maps without any detection of underlying cerebral vascular occlusion.

Noteworthy, in our cases, signs of effective ISB prior to general anesthesia were at this time point not accompanied by symptoms of CNS toxicity. This observation is surprising at first glance, as the majority of adverse events is known to occur shortly after the injection of local anesthetic. However, a review of published cases on the clinical presentation of local anesthetic systemic toxicity revealed that cardiovascular and CNS dysfunction can have a delayed onset of greater than one hour after injection [57]. This phenomenon might be explained by the comparatively low lipophilicity of lidocaine and ropivacaine [58,59], leading to a slow passage across the blood-brain barrier.

Toxic effects of local anesthetic on the CNS have been well characterized in preclinical studies in recent years. In these in vitro studies, all local anesthetics had neurotoxic effects, while the degree of neurotoxicity of local anesthetic was demonstrated to be concentration- or dose-dependent. Moreover, all drugs induced similar rates of early apoptosis at low concentrations, whereas at high concentrations, late apoptotic or necrotic cell death predominated [60,61]. These observations might explain the variable extent of cerebral lesions detected in imaging of our patients. The extent of lesions varied from no detectable structural lesions in patient 1 to extensive cortical grey matter and basal ganglia lesions in patient 2 and 3.

Interestingly, in vitro determination of toxicity induced by various local anesthetics revealed high toxicity of lidocaine and ropivacaine in neuronal cell lines [62,63]. The neurotoxic effect was mainly promoted by mitochondrial dysfunction at clinically relevant concentrations for both lidocaine and ropivacaine [60,61]. However, translation from preclinical studies to the clinical situation needs to be considered with precaution, given that the predominant share of the experimental studies available have been performed in vitro using cell cultures. Taking this into account, it is not entirely clear to what extent apoptotic and other cellular mechanisms seen in in vitro studies effectively contribute to clinical manifestation of local anesthetic neurotoxicity. Nevertheless, several clinical case reports and observational studies have shown that lidocaine and ropivacaine can induce a broad variation of severe neurotoxic side effects after intravascular injection or repeated use [29,30,55,64,65,66,67]. One of these neurotoxic side effects is a change of cerebral electrical activity observed on EEG. After unintentional intracarotid injections of lidocaine during carotid endarterectomy, both a temporary decrease in cerebral electrical activity as well as brief bursts of epileptogenic spikes have been described [67]. Epileptic seizures are a well-known complication of regional anesthesia. Interestingly, the incidence of epileptic seizures is far higher in interscalene and supraclavicular than in axillary blocks (seizure rate per 1,000 procedures 7.6 and 7.9 vs. 1.2 respectively) [53]. Arteries supplying the brain are in very close proximity to the injection trajectories of the interscalene and the supraclavicular, but not the axillar approach. This indicates that a relevant proportion of periprocedural epileptic seizures are triggered by an unintentional intraarterial injection of the anesthetics [53]. Epileptic seizures are induced by local anesthetic by blocking inhibitory synapses more strongly than excitatory synapses [68]. In our case study, EEG demonstrated both a decrease and an increase in cerebral electrical activity encompassing moderate to severe slowing in the affected hemisphere in all patients and additional seizure activity with progression to non-convulsive status epilepticus in patient 2.

Our case study has some limitations. One is the small number of cases and some variability of clinical characteristics, which prohibits statistical conclusions and therefore generalization of the results. Moreover, there is a selection bias for critical neurologic morbidity as patients were admitted to our hospital from referring hospitals. Furthermore, the blood concentrations of the local anesthetics ropivacaine and lidocaine were not assessed. However, the symptomatology of a local anesthetic-induced toxic hemisphere syndrome seen in the three cases and presented here has not previously been reported. Thus, this case series describes and highlights this hitherto unconsidered complication.

## 5. Conclusions

To the best of our knowledge there is no case series specifically describing patients with brain lesions induced by local anesthetics after ISB. We here report three cases of a local anesthetic-induced toxic left hemisphere syndrome caused with reasonable suspicion by local anesthetics used for ISB with an only partial improvement in functional outcome after hospital discharge. The severity of neurological impairment following peripheral nerve block observed in our patients should not degrade the value of this effective method of providing supplementary analgesia in the surgical setting. It rather should stimulate discussion around the neurotoxic potency of local anesthetics and emphasize that regional anesthetic techniques require appropriate postoperative clinical monitoring. In patients with new postoperative CNS symptoms following ISB, in addition to an ischemic or ictal etiology, a toxic effect of local anesthetics should be considered.

## Figures and Tables

**Table 1:** Demographic and clinical characteristics of patients with toxic hemisphere syndrome following interscalene brachial plexus block.

	Patient 1	Patient 2	Patient 3
**Demographics**			
Age (y)	52	52	71
Sex	M	M	M
Pre-existing comorbidities	MS	HT	none
Surgical procedure	Arthroscopy	Arthroscopy	Arthroscopy
Underlying disease, affected side	RCI, left	RCI, left	RCI, left
**Brachial plexus block**			
Approach, side	ISB, left	ISB, left	ISB, left
Local anesthetic	LDC, RPV	LDC, RPV	LDC, RPV
**Neurological symptoms and signs on admission**			
Consciousness impairment	yes	yes	yes
Hemisphere syndrome, side	yes, left	yes, left	yes, left
Seizure activity	yes	yes	no
Slow wave activity in affected hemisphere	yes	yes	yes
**Severity scores on admission**			
NIHSS	24	25	16
GCS	8	8	10
SAPS II	54	41	29
**Complications during hospitalisation**			
Status epilepticus	no	yes	no
Ischemic and/or hemorrhagic complications	no	no	no
Aspiration pneumonia due to dysphagia	yes	yes	no
**Treatment of toxic hemisphere syndrome**			
No. of anticonvulsants needed to control seizures	2	2	0
Need for mechanical ventilation	yes	no	no
Length (d) of hospital stay	19	9	10
**Persisting neurological symptoms and signs at discharge**			
Consciousness impairment	no	no	no
Hemisphere syndrome	yes, partial	yes, partial	yes, partial
Seizure activity	no	no	no
Slow wave activity in affected hemisphere	yes, partial	yes, partial	no

M = Male; MS = Multiple sclerosis; HT = Hypertension; RCI = Rotator cuff injury; ISB = Interscalene brachial plexus block; LDC = Lidocaine; RPV = Ropivacaine; ICU = Intensive Care Unit; GCS = Glasgow Coma Scale; NIHSS = National Institutes of Health Stroke Scale; SAPS = Simplified Acute Physiology Score.

**Table 2:** Overview of severity scores assessed in patients with toxic hemisphere syndrome during the observation period.

			Patient 1	Patient 2	Patient 3	Median
**Severity scores on admission**						
NIHSS			24	25	16	24
GCS			8	8	10	8
SAPS II			54	41	29	41
**Severity scores at discharge**						
NIHSS			10	17	7	10
GCS			14	14	15	14
GOS			3	3	4	3
**Severity scores after follow-up**						
NIHSS			2		0	1
GCS			15		15	15
GOS			4	1	5	4

NIHSS = National Institutes of Health Stroke Scale; GCS = Glasgow Coma Scale; SAPS = Simplified Acute Physiology Score; GOS = Glasgow Outcome Scale.

## Data Availability

Data are available upon reasonable request from the authors.
